# Progress in High Intensity Focused Ultrasound Ablation for Fertility Preservation Therapy of Uterine Fibroids and Adenomyosis

**DOI:** 10.1007/s43032-024-01745-y

**Published:** 2024-11-12

**Authors:** Guorui Zhang, Lei Li, Mengyuan Sun, Xin Yu

**Affiliations:** Department of Obstetrics and Gynecology, National Clinical Research Center for Obstetric & Gynecologic Diseases, Peking Union Medical College Hospital, Chinese Academy of Medical Sciences and Peking Union Medical College, No. 1, Shuaifuyuan, Wangfujing, Beijing, 100730 China

**Keywords:** High intensity focused ultrasound, Fibroid, Adenomyosis, Fertility preservation, Pregnancy outcome

## Abstract

High intensity focused ultrasound (HIFU) is an effective and safe non-invasive treatment method, widely used in the treatment of uterine fibroids and adenomyosis in the field of gynecology. The side effects in HIFU is low in incidence and mild. HIFU can significantly alleviate the symptoms of patients, reduce lesion volumes, improve quality of life, and has good cost-effectiveness. HIFU can accurately ablate the uterine fibroids and adenomyosis lesions, without destroying normal myometrium and endometrium, and thus HIFU is a promising alternative to myomectomy in uterine fibroids patients with fertility desire. Several studies have shown that in terms of ovarian endocrine function protection, HIFU treatment is superior to uterine artery embolization, and similar to myomectomy. Existing limited researches show that patients with uterine fibroids have a favorable pregnancy rate and live birth rate, as well as a lower natural abortion rate after HIFU treatment. Pregnancy rate after HIFU treatment for uterine fibroids is not lower than myomectomy, and higher than uterine artery embolization. HIFU may have significant advantages in shortening pregnancy interval compared with myomectomy. However, the proportion of cesarean section delivery after HIFU treatment is relatively high, and gestational uterine rupture after HIFU treatment exist in literature. Higher quality clinical data is needed to confirm the pregnancy outcomes and safety after HIFU treatment in future.

## Graphical Abstract

High intensity focused ultrasound (HIFU) can significantly alleviate the symptoms of patients, reduce lesion volumes, improve quality of life, and has good cost-effectiveness. HIFU provides great possibility for patients to preserve uterus and fertility function. Existing limited data suggests that HIFU treatment has a relatively high pregnancy rate and live birth rate, and low pregnancy complications rate. Higher quality clinical data is needed to confirm the pregnancy outcomes and safety after HIFU treatment.

## Background

High intensity focused ultrasound (HIFU) ablation is a non-invasive treatment technique and it is an alternative to traditional surgical local resection [1]. It utilizes the characteristics of the penetrability, aggregability and energy deposition of ultrasound, so that the ultrasound beam is focused and projected on the target tissue in human body. Then the tissue is instantly heated to above 60℃, leading to coagulation necrosis [2]. In HIFU treatment, the range of coagulation necrosis can be restricted within 1 to 3 mm to the boundaries of target lesions, causing damage to the target tissues with almost no impact on surrounding normal tissues, and thus protecting the structure and function of surrounding organs and tissues [3]. HIFU has the advantages of accuracy, adaptability, non-invasion and repeatability. The 20 years’ domestic and international clinical practice has confirmed that HIFU is an effective and safe non-invasive treatment method, widely used in the treatment of uterine fibroids and adenomyosis in the field of gynecology [4, 5]. According to the different image guidance methods, HIFU can be divided into ultrasound imaging-guided HIFU (USgHIFU) and magnetic resonance imaging-guided focused ultrasound surgery (MRgFUS) [6].

HIFU has the advantages of accuracy, adaptability, non-invasion and repeatability, making it a promising alternative to myomectomy in uterine fibroids patients with fertility desire. Pregnancy after HIFU treatment is currently a hot topic in the field of uterine fibroid treatment. At present, there are some observational cohort studies in the literature reporting pregnancy related issues after HIFU treatment for uterine fibroids and adenomyosis, elaborating on obstetric outcomes such as pregnancy rate, live birth rate, and spontaneous abortion rate. There are also some studies on delivery methods and pregnancy safety. However, overall, clinical data on pregnancy after HIFU treatment for uterine fibroids and adenomyosis is limited and the level of evidence is low. This review focuses on the progress of high-intensity focused ultrasound ablation in treating uterine fibroids and adenomyosis patients with fertility desires, and summarizing their fertility outcomes and complications after high-intensity focused ultrasound ablation.

## Main Text

### HIFU in the Treatment of Uterine Fibroids with Fertility Preservation

Uterine fibroids are the most common gynecological benign tumors in women, increasing the risk of infertility and spontaneous abortion, as well as the risk of pregnancy complications such as premature delivery, abnormal presentation of the fetus, and postpartum hemorrhage [7–10]. Uterine fibroids affect the fertility and pregnancy safety of women in childbearing age [11, 12]. For women with fertility requires, myomectomy with different approaches is currently the mainstream treatment. However, surgical operation not only treats fibroids, but also brings fertility damage [13]. In addition, postoperative scar uterus and pregnancy interval requirements further damage the fertility potential of older women and increase the risk of complications in pregnancy [14, 15]. HIFU can precisely ablate the uterine fibroid lesions, causing almost no damage to the normal myometrium and endometrium, and exerting no impact on ovarian reserve [16–18]. Thus, HIFU has broad application prospects in preserving fertility function in uterine fibroids [19].

#### The Effectiveness and Safety of HIFU in the Treatment of Uterine Fibroids

The efficacy of HIFU in the treatment of uterine fibroids is not inferior to that of traditional surgery, significantly alleviating symptoms. The safety of HIFU is significantly better than surgery, improving the life quality of patients. In 2002, researchers in China first reported the preliminary results of a clinical study of HIFU in the treatment of symptomatic uterine fibroids. In October 2004, MRgFUS was approved by the US Food and Drug Administration for the treatment of uterine fibroids [20]. In 2011, with the support of the Ministry of Science and Technology, 20 hospitals in China were organized to carry out a prospective multi-center and non-random parallel controlled study on HIFU in uterine fibroids [21]. 1353 patients with HIFU treatment, 472 patients with hysterectomy and 586 patients with myomectomy were enrolled. The results showed that the life quality score (measured by uterine fibroid symptom quality-of-life questionnaire) of patients in the HIFU group was higher than that in the surgery group at 6 months (82.49 vs. 80.44) and 12 months (85.84 vs. 83.45) visit. HIFU showed advantages in physiological function improvement, bodily pain relief, vitality restoration and emotional recovery. Mean duration of hospital stay was 3.6 days for HIFU, 10.5 days for hysterectomy and 9 days for myomectomy. The average cost of treatment was lower for HIFU than surgical therapy. The incidence of major adverse events in the HIFU group was lower (3 cases in the HIFU group, with an incidence rate of 0.22%; 133 cases in the surgery group, with an incidence rate of 12.6%), as well as the incidence of minor adverse events (335 cases in the HIFU group, with an incidence rate of 24.8%; 719 cases in the surgery group, with an incidence rate of 68.0%). All three major adverse events in the HIFU group were second-degree skin burns, while major adverse events in the surgery group included hemorrhage, infection, thromboembolic events and injury to the bladder. The minor events recorded in the HIFU group included pain, weakness, or numbness in the lower limbs, back or perineum, hematuria, nausea and dizziness. This study indicates that efficacy of HIFU is not inferior to the traditional surgery in the treatment of uterine fibroids, and the safety of HIFU is significantly better than surgery. HIFU significantly alleviates symptoms, improves patient quality of life, significantly reduces hospital stay, and saves medical costs, with minimal trauma. It is a promising treatment strategy for uterine fibroids [22].

Evidences have demonstrated that HIFU can significantly improve symptoms related to uterine fibroids, leading to an improvement of health-related quality of life [20, 23]. In a study enrolling 55 women with symptomatic uterine fibroids [24], a significant reduction of the symptom severity scale was observed at 6 weeks and 12 months after HIFU treatment (49.9 ± 19.4 at baseline vs. 42.2 ± 20.1 at 6 weeks and 23.6 ± 12.7 at 12 months after treatment, *p* < 0.001), correlating with a significant improvement of Health-related Quality of Life (HRQL) (52.5 ± 22.7 at baseline vs. 59.8 ± 22 at 6 weeks and 77.9 ± 17.3 at 12 months after treatment, *p* < 0.001). A study in Korea [25] enrolling 272 patients of uterine fibroids, showed that the symptom severity scale reduction rates were 55.58%, 52.76%, and 50.39% at 3, 6, and 12 months follow-up, respectively. What is more, HIFU can reduce fibroid volume [26]. The uterine fibroid volume reduction rates (%) were 58.08%, 66.18%, and 77.59% at 3, 6, and 12 months after treatment, respectively [25].

Results of meta-analysis support that HIFU is as effective as myomectomy or uterine artery embolization in the treatment of uterine fibroids. A meta-analysis of 21 studies [17] showed that compared with the operation group, the patients in the HIFU group had higher health-related quality of life score (MD = 2.25, 95% CI: 1.15–3.35), similar symptom severity score, and no difference in re-intervention rate (RR = 1.65, 95% CI: 0.59–4.57). Compared with uterine artery embolization, there was no significant difference in health-related quality of life score and symptom severity score in the HIFU group, but the re-intervention rate was higher (RR 4.06, 95% CI: 2.47–6.69) in the HIFU group [17]. Another meta-analysis of 18 studies [27] showed that compared with myomectomy, the hospitalization time in HIFU group was shorter (MD -4.70, 95% CI: 7.46–1.94), and the incidence of complications was lower, but the rate of re-intervention was higher (OR = 4.05, 95% CI: 1.82–8.9). However, a meta-analysis including 207 patients from 4 studies [28] showed that MRgFUS might not be superior to that of uterine artery embolization in reducing symptom severity score, improving health-related quality of life, reducing re-intervention rate, and rate of adverse reactions in uterine myoma.

The cost–utility of HIFU treatment in comparison to conventional hysterectomy was less costly. Babashov [29] reported that compared with hysterectomy, MRgFUS was cost-effective with an incremental cost of $39,250 per additional per quality-adjusted life-year gained, whereas uterine artery embolization had an incremental cost-effectiveness ratio of $46,480. A study in China [30] showed that HIFU was less costly than open hysterectomy in adenomyosis, and the quality adjusted life year for patients treated with HIFU was $5256.48, whereas it was $7510.03 for patients treated with open hysterectomy. The long-term cost-effectiveness analysis [31] of MRgFUS and standard minimally invasive fibroid treatments in Netherlands (Trial Register NL8863) is ongoing, and primary outcomes are quality of life 24 months after treatment and costs of treatment including direct health care costs, loss of productivity, and patient costs. However, some scholars worried that he hidden costs of training staff, extended learning curve and lack of international guidelines mean that HIFU faced many logistical, systemic and financial challenges [32].

#### The Influencing Factors and Predictive Methods of Efficacy of HIFU on Uterine Fibroids

The characteristics of fibroids in magnetic resonance imaging (MRI) and contrast-enhanced ultrasound examination affect the therapeutic effect of HIFU, and may become a method to predict the therapeutic effect. According to the signal characteristics of T2-weighted MRI, uterine fibroids can be divided into Funaki 1–3 types. A single-center study in Germany [33] showed that the proportion of non-perfusion areas in Funaki 1 and 2 types of fibroids after HIFU treatment was higher (*p* = 0.023), suggesting that HIFU induced better ablation effect in Funaki 1 and 2 types fibroids. Radiological parameters based on T2-weighted MRI could predict the regeneration of residual uterine fibroids within one year after HIFU treatment [34]. Wang [35] evaluated the predictive significance of contrast-enhanced ultrasound quantitative perfusion parameters for HIFU treatment response in patients with uterine fibroids. The results showed that the arrival time, peak time and enhancement time of the group with 70% or higher unfused volume ratio were longer than those of the group with less than 70% unfused volume ratio, while the average enhancement intensity and enhancement rate were lower [35]. The author believed that the quantitative parameters of contrast-enhanced ultrasound might be helpful to evaluate the ablation effect of HIFU treatment in uterine fibroids [35]. For patients with poor heating efficacy, the main causes for the diminished heating were likely the high local perfusion and ultrasound attenuation due to the deep location of the myoma [36].

### HIFU in the Treatment of Adenomyosis with Fertility Preservation

Adenomyosis features the presence of endometrial glands and stroma with growth function in the myometrium, accompanied by compensatory hypertrophy and proliferation of surrounding myometrium cells [37]. Clinically, adenomyosis is characterized by progressive aggravated dysmenorrhea, increased menstrual volume and prolonged menstrual period [38]. It is a common female reproductive system disease that seriously affects fertility and quality of life [39–41]. However, the treatment of adenomyosis is challenging, especially for patients with fertility desire. The patient’s age, severity of symptoms, desire for future fertility, and related pelvic lesions (such as uterine fibroids and endometriosis) are important considerations for making treatment decisions. Current treatments for adenomyosis include surgical therapy and conservative medical treatment [42, 43]. Gonadotropin-releasing hormone analogue (GnRH-a) and adenomyomectomy are the main treatment options for adenomyosis with fertility desire [42, 44]. HIFU is a novel, noninvasive treatment for adenomyosis, which may relieve dysmenorrhea and menorrhagia by inducing inflammatory factors and downregulating the expression of vascular endothelial growth factors in adenomyotic lesions [45–48]. HIFU has been used to treat both focal and diffuse adenomyosis [49, 50].

#### The Effectiveness and Safety of HIFU in the Treatment of Adenomyosis

HIFU can reduce the volume of adenomyosis lesions, alleviate symptoms including dysmenorrhea and increased menstrual volume, and improve patients’ quality of life [51–53]. A single-center study reported by Li [45], which enrolled 485 patients with adenomyosis who received HIFU treatment, showed that the dysmenorrhea severity pain score and average menorrhagia severity score decreased significantly at each follow-up time point after HIFU ablation. The results of a meta-analysis [54] showed that after HIFU treatment for adenomyosis, a significant relief in dysmenorrhea was observed at 3 months, a significant improvement in patients’ quality of life was observed at 6 months, and a significant reduction in uterine volume was observed at 12 months.

HIFU has a high symptom relief rate in different types of adenomyosis. In a retrospective analysis [55] of 321 patients with adenomyosis who received HIFU treatment, adenomyosis was classified into internal, external, full thickness, and intramural adenomyomas based on the relationship between adenomyosis lesions and uterine structure. Asymmetric and symmetrical adenomyosis were classified based on the degree of involvement of the uterine myometrium. The results showed the dysmenorrhea relief rates of patients with asymmetric internal, symmetric internal, asymmetric external, asymmetric full thickness, symmetric full thickness, and intramural adenomyosis were 68.3%, 62.1%, 54.7%, 64.1%, 60%, and 100% at 18 months follow-up after treatment, and the menstrual volume reduction rates were 68.3%, 51.6%, 51.0%, 55.5%, 57.2%, and 100%, respectively [55]. In addition, HIFU is safe for the treatment of diffuse adenomyosis, and controlling the ablation zone is crucial to ensure patients’ safety. A retrospective cohort study [56] including 260 patients with diffuse adenomyosis (Group D) and 157 patients with focal adenomyosis (Group F), the non-perfused volume ratio (NPVR) of Group D was significantly lower than that of Group F (*p* < 0.05). 97 patients (23.3%) received nominal therapy due to complications (B grade), among them, there were 62 cases (23.8%) in Group D and 35 cases (22.3%) in Group F [56]. No significant difference was found between the two groups (*p* > 0.05) and neither of the reported complications of grade C-F occurred within the two groups [56]. At present, there is a lack of randomized controlled clinical trials of HIFU and other uterus-preserving treatments for adenomyosis. HIFU is a new and promising treatment option for patients with adenomyosis, but its efficacy, safety, cost-effectiveness, and fertility outcomes still need to be evaluated through randomized controlled trials in future.

The combination of HIFU and GnRH-a or levonorgestrel intrauterine release system (LNG-IUS) may have better clinical efficacy [57–59]. The clinical effect of HIFU combined with GnRH-a is superior to HIFU alone. A meta-analysis included 766 patients from 9 studies [60] demonstrated that compared with the HIFU group, the HIFU combined with GnRH-a group had a higher rate of uterine volume reduction (MD 7.51), a smaller volume of adenomyosis lesions (MD 4.11), lower dysmenorrhea visual analogue scale score (MD 1.27), and lower menstrual volume score (MD0.88). The postoperative CA125 level was lower (SMD 0.31), and the recurrence rate was lower in the HIFU combined with GnRH-a group than that of the HIFU group alone (RR 0.28) [60]. The combination of HIFU and GnRH-a or LNG-IUS has a better effect on alleviating dysmenorrhea symptoms in patients with severe adenomyosis [61]. A single-center study reported by Li [45] included 1982 adenomyosis patients treated by HIFU. The results showed that after 6 months and 3 years of treatment, the efficacy of HIFU combined with LNG-IUS, HIFU combined with GnRH-a and LNG-IUS was significantly better than that of HIFU alone and HIFU combined with GnRH-a(*p* < 0.05) [45]. A retrospective study of 243 patients by Xu [62] showed that there was no difference in dysmenorrhea improvement among HIFU group, HIFU combined with GnRH-a group and HIFU combined with LNG-IUS group within 6 months of treatment, but after 12 months, the improvement rates of dysmenorrhea in the three groups were 77.38%, 79.52% and 96.05% respectively. The researchers believed that HIFU alone could effectively alleviate dysmenorrhea symptoms in the short term, but the combination of HIFU and LNG-IUS could improve the treatment effect over a longer period of time [62]. Another meta-analysis by Zhao [63] enrolled 1861 patients in 13 studies, the results showed compared with the HIFU group, the HIFU and LNG-IUS group had more pronounced reduction in uterine volume at 6 months (MD 29.04) and 12 months (MD 22.10) after HIFU, and the HIFU and LNG-IUS group had lower visual analogue scale scores for dysmenorrhea at 3 months(MD 1.68), 6 months(MD 1.69), and 12 months (MD 1.30) after HIFU ablation. However, due to the limited sample size and mostly retrospective studies, high-quality evidence of its long term efficacy is still insufficient.

#### The Influencing Factors and Predictive Methods of Efficacy of HIFU on Adenomyosis

The general clinical characteristics of patients are related to the therapeutic effect. A retrospective study [64] of 230 patients found that older women (OR 0.342) were more likely to achieve clinical success. Women with long-term treatment success were significantly older at the time of treatment (46.3 vs. 43.6 years, *p* = 0.02) [65].In addition, lower body mass index (OR 1.221) and higher sound power (OR 0.992) were associated with a lower recurrence risk [64]. After treatment, patients with a higher proportion of non-perfusion areas (OR 0.964) showed more significant symptom relief [64]. These factors are helpful to select suitable HIFU treatment patients and predict the persistence of symptom relief.

The imaging characteristics of magnetic resonance adenomyosis may be used to predict the therapeutic effect [66]. Multiple factors such as the enhancement type of the adenomyotic lesion, volume of the adenomyotic lesions, number of hyperintense foci on T2WI, location of the uterus, location of adenomyotic lesions, thickness of the abdominal wall and distance from the skin to the adenomyotic lesions contribute to the efficacy of HIFU [49]. Hypo-intense fibroids on T2-weighted MRI was associated with higher success rates and could be used to predict success rates on the basis of their presence or absence as pre-treatment parameters [65, 67]. A retrospective study of 245 cases showed that the enhancement type on T1WI, the signal intensity on T2WI, the volume and location of adenomyosis lesions, the number of high signal spots, abdominal wall thickness, and distance from skin to the ventral side of adenomyosis lesions could all be used as predictive indicators for efficacy of HIFU treatment [68]. The number of high signal foci on T2-weighted MRI was a factor that affected the ablation rate of adenomyosis patients and the clinical efficacy of HIFU treatment in patients with adenomyosis [69]. A prospective study included 102 patients with adenomyosis and found that patients with less than 5 high signal lesions on T2WI had a higher ablation rate than those with more than 5 high signal lesions (*p* < 0.05) [70]. What’s more, patients with pelvic endometriosis, adhesions between the bowel and the uterus, or an abdominal surgical scar wider than 10 mm, are not suitable for HIFU treatment.

The level of CA125 has certain predictive value on predicting the efficacy of HIFU in adenomyosis. A retrospective study [71] of 502 adenomyosis patients found that patients with elevated preoperative CA125 level had a higher risk of symptom recurrence after treatment (OR 1.002), and the recurrence time (38.5 months) in the group with preoperative CA125 levels > 35 U/ml was shorter than that in the group with CA125 levels ≤ 35 U/ml (44.5 months, *p* = 0.001).

### Fertility Issues after HIFU Treatment in Fibroids and Adenomyosis

HIFU can accurately ablate the uterine fibroids and adenomyosis lesions, without destroying normal myometrium and endometrium, and has no effect on ovarian endocrine function, which provides great possibility for patients to preserve uterus and fertility function. Nevertheless, at present, hysteroscopic, laparoscopic and abdominal myomectomy are the most commonly used treatment methods for uterine fibroids in women with fertility desire in clinical practice, though a long postoperative contraception period is needed [72]. HIFU is a promising alternative to myomectomy in uterine fibroids patients with fertility desire. However, there is a lack of large sample data on pregnancy outcome and pregnancy safety after HIFU ablation. More controlled trials comparing reproductive outcomes after HIFU is necessary to provide sufficient evidence on the treatment recommendations for uterine fibroids and adenomyosis patients with fertility desire.

#### HIFU Does not Affect Ovarian Reserve Function

Several studies have shown that HIFU treatment has no significant effect on ovarian reserve [73]. Otonkoski [18] reported 74 fibroids patients who underwent MRgFUS, with a median anti-Müllerian hormone (AMH) level of 1.20 ug/L (range 0.1–7.75 ug/L) before treatment and 1.23 ug/L (range 0.1–8.51 ug/L) after treatment. The difference was not statistically significant, and the location of the fibroids and treatment energy were not related to changes in ovarian function. Another prospective study [74] enrolling 79 symptomatic uterine fibroids and adenomyosis patients showed that the median AMH levels were 2.11 ± 2.66ug/L and 1.84 ± 2.57ug/L before and 6 months after HIFU ablation, respectively. There was no significant difference in AMH levels between the two time points (*p* > 0.05).

In terms of ovarian endocrine function protection, HIFU treatment is superior to uterine artery embolization, and similar to myomectomy. Laughlin [75] reported that the median absolute change in AMH levels was significantly larger in the uterine artery embolization group than in the HIFU group (-0.6 vs. -0.2, *p* = 0.03) after 24 months of follow-up. Cui [76] compared the changes in ovarian hormone levels and the resistance index (RI) and the pulsatility index (PI) of the uterine arterial blood flow after HIFU treatment and myomectomy. The results showed no significant changes in PI, RI, estradiol, follicle stimulating hormone and luteinizing hormone levels before and after 6 months of treatment between the two groups and within the same group (*p* > 0.05).

#### Pregnancy Outcome after HIFU Treatment

Existing limited researches show that patients with uterine fibroids have a favorable pregnancy rate and live birth rate, as well as a lower natural abortion rate after HIFU treatment.

The pregnancy rate is high after HIFU treatment for uterine fibroids. A prospective study [77] enrolled 174 patients who received HIFU treatment and had pregnancy plans. During a 76 months’ follow-up period, 81 patients had 88 pregnancies, with a pregnancy rate of 47%. The pregnancy rate in other three studies was 69.3% (131/189, follow-up time 3 years) [78], 19.2% (78/406, follow-up time unknown) [79] and 16.0% (4/25, follow-up time 1 year) [80]. After combining the data from these four studies, the pregnancy rate of 838 women was 36% [81]. Pregnancy rate after HIFU treatment for uterine fibroids is not lower than that after myomectomy, and higher than that after uterine artery embolization. Wu [82] retrospectively summarized 320 patients receiving HIFU treatment, and results suggested that after a median of 5 years’ follow-up, the pregnancy rate did not significantly differ between the HIFU groups and laparoscopic myomectomy group (68.4% vs. 66.7%, *p* = 0.730). In Yan’s meta-analysis [17], the postoperative pregnancy rates were not significantly different (RR = 1.01, 95% CI: 0.90–1.13) in the HIFU group and the surgical group; the postoperative pregnancy rate in the HIFU group was significantly higher than the uterine artery embolization group (RR = 17.44, 95% CI: 2.40–126.50).

The live birth rate is high after HIFU treatment for uterine fibroids, and the spontaneous abortion rate is low. In Liu’s prospective study [77], the rate of spontaneous abortion among all pregnancies was 10% (9/88), and 84% (74/88) of the pregnancies resulted in live births. Zou [79] retrospectively reported that spontaneous abortion occurred in 3 patients out of 80 pregnancies, with a spontaneous abortion rate of 3.8%, and live births were achieved in 71 patients(88.8%). The live birth rate of all reported pregnancies was 91%, and the miscarriage rate was 4–15% in a summary report [81]. In Wu’s retrospective study [82], rate of spontaneous abortion did not significantly differ between the HIFU groups and laparoscopic myomectomy group (5.0% vs. 5.9%, *p* = 0.814).

What is more, HIFU may have significant advantages in shortening pregnancy interval [17] compared with myomectomy. The median time interval from HIFU treatment to pregnancy ranges from 4 to 16 months. Zou [79] reported that the average time interval from HIFU treatment to pregnancy was 5.6 months (range 1–18 months). Specifically, the time interval in six patients (7.5%) was equal to or less than 3 months, in 13 patients (16.3%) between 3 and 6 months, and in 61 patients (76.2%) over 6 months. In Wu’s retrospective study [82], interval from fibroids treatment to the following pregnancy was significantly shorter in the HIFU group than in the laparoscopic myomectomy group (13.6 ± 9.5 months vs. 18.9 ± 7.3 months, *p* < 0.05).

The pregnancy outcome of submucosal uterine fibroids after HIFU treatment is optimistic, not inferior to hysteroscopic myomectomy. Li [83] retrospectively summarized the pregnancy outcome of patients with a solitary submucosal fibroid. In the study, 7 patients (7/11, 63.6%) in the HIFU group and 24 patients (24/48, 50%) in the hysteroscopic myomectomy group got pregnant. There was no significant difference in pregnancy rate. In the HIFU group, 6 (85.7%) patients got live births, and 1 (14.3%) patient underwent spontaneous abortion; while in the hysteroscopic myomectomy group, 22 (91.7%) patients got live births, and 2 (8.3%) patients underwent spontaneous abortion.

Limited data supports that the pregnancy rate and live birth rate of adenomyosis patients after HIFU treatment are high. Wei [84] reported that 50 (38.7%) out of 129 adenomyosis patients became pregnant after HIFU during a median of 26 months of follow-up. Among them, 26 cases with live births, and 18 cases with spontaneous abortions were recorded at follow up endpoint. Zhou [85] retrospectively reported 54 patients out of 68 patients with adenomyosis got pregnancy after HIFU ablation, and 21 patients had live births, with a median time interval from HIFU to pregnancy of 10 months(range 1–31 months). In Zhou’s study [85], 20 of the 54 pregnancies had spontaneous abortion, and there was no significant difference between the preoperative and postoperative spontaneous abortion rate (23/54, 42.6% vs. 20/54, 37.0%; *p* > 0.05) in patients with adenomyosis receiving HIFU ablation. The different types of adenomyosis affect the pregnancy outcome after HIFU treatment. The pregnancy rate after HIFU treatment for patients with internal adenomyosis, external adenomyosis, intramural adenomyosis, and full-thickness adenomyosis was 59.6%, 22.9%, 17.6%, and 32.0%, respectively [84]. Patients with adenomyotic lesions located in the posterior wall of the uterus had a higher pregnancy rate than those with lesions located in the fundus of the uterus [84].

HIFU may provide possibilities of increased pregnancy rate in patients with adenomyosis complicated with infertility, compared with adenomyomectomy. Huang [86] retrospectively analyzed the pregnancy outcome of 93 cases of adenomyosis with infertility. Results showed pregnancy rate in the HIFU group (26/50, 52%) was higher than the laparoscopic adenomyomectomy group (13/43, 30.2%; *p* = 0.034). However, the live birth rates were not significant different between the two group (18/50, 36.0% vs. 12/47, 23.9%; *p* = 0.405). Among them [86], spontaneous abortion occurred in 3 patients in the HIFU group, and 1 patient in the laparoscopic excision group. Notably, in Huang’s study [86], 2 patients with a history of spontaneous abortion had pregnancy and live births after HIFU treatment. Whether HIFU improves pregnancy rate and live birth rate in adenomyosis patients complicated with infertility and spontaneous abortion, it still needs further exploration.

#### Mode of Delivery after HIFU Treatment

In existing literature reports, the proportion of cesarean section delivery after HIFU treatment is relatively high. In terms of mode of delivery, among the three studies with larger samples, the cesarean section rate were 72% (52/72) [77], 72%(67/93) [78] and 81%(56/71) [79], respectively. Concerning the indications for cesarean section, 13% were due to obstetric reasons, including premature rupture of membranes, fetal distress, breech position, head pelvic imbalance or oligohydramnios, while the rest were social factors. In Liu’s study, 69% (36/52) of the indications of cesarean section were social factors, with patients worried about uterine rupture in 15 patients and obstetricians recommending a cesarean section merely due to HIFU history in 11 patients [77]. In Gu’s retrospective study [67], among 90,685 deliveries in three years, 40 patients (0.044%) had previously received HIFU, and 37 patients (92.5%) were eligible for admission to trial of labor after HIFU. Among those patients, 36 patients undergoing cesarean section, 11 patients for obstetric factors, and 25 patients for obstetricians’ recommendations (21 patients had received a recommendation from obstetricians who took a conservative approach to managing labor in pregnant women with a history of HIFU ablation treatment). A research reported that the cesarean section rate after HIFU treatment was lower than that after myomectomy. In Wu’s retrospective study [82], the rate of cesarean section was significantly lower in the HIFU group than in the laparoscopic myomectomy (41.6% vs. 54.9%, *p* < 0.05).

Vaginal delivery after HIFU treatment is relatively safe. In Liu’s prospective study [77], 21 (80.8%) out of 26 patients who were scheduled for vaginal delivery after HIFU treatment, were successful. Zou [79] retrospectively reported 15 cases of vaginal deliveries among 71 live births. No server complications, including uterine rupture, postpartum hemorrhage and placental implantation, were reported in Liu’s and Zou’s studies. Several researches have demonstrated that obstetricians and patients are not confident in vaginal birth after HIFU treatment [67]. One important reason is the current lack of data on complications of vaginal delivery after HIFU, including postpartum hemorrhage, uterine atony and uterine rupture. Vaginal delivery after HIFU is a new clinical practice, it needs more time for obstetricians and gynecologists to fully understand HIFU, as well as numerous studies to confirm the safety of vaginal birth after HIFU and strategies to control complications.

There are few reports on delivery modes after HIFU treatment for adenomyosis. In Huang’s study [86], 6 cases of vaginal delivery and 12 cases of cesarean section were reported in adenomyosis patients after HIFU treatment. Unfortunately, the researcher did not clarify the indications for cesarean section.

#### Pregnancy Safety after HIFU Treatment

There is still a lack of data from large-scale clinical studies on pregnancy safety after HIFU. In a literature review that included 14 studies, a total of 366 pregnancies after USgHIFU were reported, including 1 case of intrauterine fetal death, 6 cases of placenta previa, and no uterine rupture; and 124 cases of pregnancy after MRgFUS, with 2 cases of placenta previa and no uterine rupture [81]. In Wu’s retrospective study [82], incidences of placenta increta (1.1% vs. 6.4%, *p* < 0.05), and placenta previa (2.8% vs. 8.7%, *p* < 0.05) were lower after HIFU compared with that after laparoscopic myomectomy, while incidences of preterm birth, fetal distress, fetal growth restriction, and postpartum hemorrhage showed no difference. In Gu’s retrospective study [67], the prevalence of postpartum hemorrhage (blood loss over 500 ml) and severe postpartum hemorrhage (blood loss over 1000 ml) in the qualified candidates for trial of labor after HIFU group was not significantly different from those in the candidates for trial of labor after cesarean group (8.8% vs. 10.5%, *p* = 0.534; 0% vs. 2.5%, *p* = 0.418).

Data on complications during pregnancy and delivery in adenomyosis patients after HIFU is limited. In Huang’s retrospective study [86], a total of 5 patients (10%) experienced complications during pregnancy and delivery, including 2(4%) cases of placenta accreta, 2(4%) cases of postpartum hemorrhage, with blood loss volumes of 1300 and 1000mL, respectively, and 1 case (2%) of premature rupture of membrane. The incidences of those complications were not different from those after laparoscopic adenomyomectomy.

However, there is a risk of uterine rupture after HIFU treatment [87, 88]. At present, 4 cases of uterine rupture during pregnancy after HIFU treatment can be retrieved from the literature. The detailed information of the 4 cases with uterine rupture was briefly summarized as follows. Li [89] reported a spontaneous uterine rupture at 38 weeks of gestation after HIFU for an anterior wall fibroid 8.2 cm in diameter, with an interval of 20 months between HIFU and pregnancy. Lai [90] reported a uterine rupture at 37 weeks of gestation in a 44-year-old patient who received HIFU treatment for fibroids and adenomyosis, with an interval of 20 months between HIFU and in vitro fertilization. Liu [91] reported a uterine rupture at 38 weeks of gestation after HIFU for an anterior wall adenomyosis sized 7.0 cm, with an interval of 8 months between HIFU and unplanned pregnancy. Wu [82] reported an incomplete uterine rupture at 36 weeks of gestation in a 38-year-old patient after HIFU for a 56 mm single intramural myoma located in the right anterior wall, with an interval of 4 months between HIFU and pregnancy. Due to only a few reported cases of uterine rupture after HIFU, the risk factors for uterine rupture are still unclear. With the increasing popularity of HIFU in the treatment of fibroid and adenomyosis, it is necessary to be alert to the risk of uterine rupture during pregnancy.

## Conclusions

HIFU has good clinical efficacy in the treatment of fibroid and adenomyosis, with mild and rare complications, making it a minimally invasive treatment method with broad application prospects. HIFU has many therapeutic advantages in fertility protection for fibroids and adenomyosis patients with fertility desires. However, there is still a lack of consensus on the treatment recommendation, patient selection, factors relating to treatment efficacy and impact on pregnancy outcome. Further research is needed on the safety of pregnancy after HIFU treatment, and prevention of maternal-fetal related complications.

## Data Availability

Not applicable.

## References

[1] Griffiths A, terHaar G, Rivens I, Giussani D, Lees C. High-intensity focused ultrasound in obstetrics and gynecology: the birth of a new era of noninvasive surgery? Ultraschall Med. 2012;33(7):E8–15. 22723041 10.1055/s-0031-1299407

[2] Izadifar Z, Izadifar Z, Chapman D, Babyn P. An introduction to high intensity focused Ultrasound: systematic review on principles, devices, and clinical applications. J Clin Med. 2020;9(2). 10.3390/jcm9020460 PMC7073974 32046072

[3] Zhang L, Wang ZB. High-intensity focused ultrasound tumor ablation: review of ten years of clinical experience. Front Med China. 2010;4(3):294–302. 21191835 10.1007/s11684-010-0092-8

[4] Lee KW. The Asian perspective on HIFU. Int J Hyperth. 2021;38(2):5–8. 10.1080/02656736.2021.1889697 34420444

[5] Stovall DW. Alternatives to hysterectomy: focus on global endometrial ablation, uterine fibroid embolization, and magnetic resonance-guided focused ultrasound. Menopause. 2011;18(4):437–44. 21701430 10.1097/gme.0b013e318207fe15

[6] Xu Y, Fu Z, Yang L, Huang Z, Chen WZ, Wang Z. Feasibility, Safety, and efficacy of Accurate Uterine fibroid ablation using magnetic resonance imaging-guided high-intensity focused ultrasound with shot sonication. J Ultrasound Med. 2015;34(12):2293–303. 26518278 10.7863/ultra.14.12080

[7] Bulun SE. Uterine fibroids. N Engl J Med. 2013;369(14):1344–55. 24088094 10.1056/NEJMra1209993

[8] Harlev A, Wainstock T, Walfisch A, Landau D, Sheiner E. Perinatal outcome and long-term pediatric morbidity of pregnancies with a fibroid uterus. Early Hum Dev. 2019;129:33–7. 30639463 10.1016/j.earlhumdev.2019.01.004

[9] Karlsen K, Schioler Kesmodel U, Mogensen O, Humaidan P, Ravn P. Relationship between a uterine fibroid diagnosis and the risk of adverse obstetrical outcomes: a cohort study. BMJ Open. 2020;10(2):e032104. 32071172 10.1136/bmjopen-2019-032104 PMC7044982

[10] Saleh HS, Mowafy HE, Hameid A, Sherif HE, Mahfouz EM. Does uterine fibroid adversely affect obstetric outcome of pregnancy? Biomed Res Int. 2018;2018:8367068. 30151390 10.1155/2018/8367068 PMC6087613

[11] Giuliani E, As-Sanie S, Marsh EE. Epidemiology and management of uterine fibroids. Int J Gynaecol Obstet. 2020;149(1):3–9. 31960950 10.1002/ijgo.13102

[12] Van Heertum K, Barmat L. Uterine fibroids associated with infertility. Womens Health (Lond). 2014;10(6):645–53. 25482490 10.2217/whe.14.27

[13] Grube M, Neis F, Brucker SY, Kommoss S, Andress J, Weiss M, et al. Uterine fibroids - current Trends and Strategies. Surg Technol Int. 2019;34:257–63. 30888674

[14] Mahalingam M, Hu M, Schointuch M, Szychowski JM, Harper L, Owen J, et al. Uterine myomas: effect of prior myomectomy on pregnancy outcomes. J Matern Fetal Neonatal Med. 2022;35(25):8492–7. 34615420 10.1080/14767058.2021.1984424 PMC10961099

[15] Kim YR, Na ED, Jung JE, Moon JH, Lee JY. Clinical features at the time of non-hysteroscopic myomectomy before pregnancy, which affect adverse pregnancy outcomes: a retrospective cohort study. BMC Pregnancy Childbirth. 2022;22(1):896. 36463110 10.1186/s12884-022-05240-7 PMC9719619

[16] Silberzweig JE, Powell DK, Matsumoto AH, Spies JB. Management of uterine fibroids: a focus on uterine-sparing interventional techniques. Radiology. 2016;280(3):675–92. 27533290 10.1148/radiol.2016141693

[17] Yan L, Huang H, Lin J, Yu R. High-intensity focused ultrasound treatment for symptomatic uterine fibroids: a systematic review and meta-analysis. Int J Hyperth. 2022;39(1):230–8. 10.1080/02656736.2022.2029956 35094613

[18] Otonkoski S, Sainio T, Mattila S, Blanco Sequieros R, Perheentupa A, Komar G, et al. Magnetic resonance guided high intensity focused ultrasound for uterine fibroids and adenomyosis has no effect on ovarian reserve. Int J Hyperth. 2023;40(1):2154575. 10.1080/02656736.2022.2154575 36535925

[19] Jeng CJ, Ou KY, Long CY, Chuang L, Ker CR. 500 cases of high-intensity focused Ultrasound (HIFU) ablated uterine fibroids and adenomyosis. Taiwan J Obstet Gynecol. 2020;59(6):865–71. 33218403 10.1016/j.tjog.2020.09.013

[20] Mahmoud MZ, Alkhorayef M, Alzimami KS, Aljuhani MS, Sulieman A. High-intensity focused Ultrasound (HIFU) in uterine fibroid treatment: review study. Pol J Radiol. 2014;79:384–90. 25371765 10.12659/PJR.891110 PMC4218899

[21] Chen J, Li Y, Wang Z, McCulloch P, Hu L, Chen W, et al. Evaluation of high-intensity focused ultrasound ablation for uterine fibroids: an IDEAL prospective exploration study. BJOG. 2018;125(3):354–64. 28421665 10.1111/1471-0528.14689

[22] Lee JS, Hong GY, Lee KH, Song JH, Kim TE. Safety and Efficacy of Ultrasound-guided high-intensity focused Ultrasound Treatment for Uterine fibroids and adenomyosis. Ultrasound Med Biol. 2019;45(12):3214–21. 31563479 10.1016/j.ultrasmedbio.2019.08.022

[23] Fischer K, McDannold NJ, Tempany CM, Jolesz FA, Fennessy FM. Potential of minimally invasive procedures in the treatment of uterine fibroids: a focus on magnetic resonance-guided focused ultrasound therapy. Int J Womens Health. 2015;7:901–12. 26622192 10.2147/IJWH.S55564 PMC4654554

[24] Tonguc T, Recker F, Ganslmeier J, Strunk HM, Pieper CC, Ramig O, et al. Improvement of fibroid-associated symptoms and quality of life after US-guided high-intensity focused ultrasound (HIFU) of uterine fibroids. Sci Rep. 2022;12(1):21155. 36476975 10.1038/s41598-022-24994-w PMC9729612

[25] Lee JS, Hong GY, Park BJ, Kim TE. Ultrasound-guided high-intensity focused ultrasound treatment for uterine fibroid & adenomyosis: a single center experience from the Republic of Korea. Ultrason Sonochem. 2015;27:682–7. 26072367 10.1016/j.ultsonch.2015.05.033

[26] Liu J, Keserci B, Rong R, Wei J, Zhang J, Wang X. Effect of the degree of magnetic resonance-guided high-intensity focused ultrasound ablation of uterine fibroid tissue on 6-month volume reduction. Int J Gynaecol Obstet. 2017;137(1):92–4. 28084010 10.1002/ijgo.12102

[27] Wang Y, Geng J, Bao H, Dong J, Shi J, Xi Q. Comparative effectiveness and safety of high-intensity focused Ultrasound for Uterine fibroids: a systematic review and Meta-analysis. Front Oncol. 2021;11:600800. 33767979 10.3389/fonc.2021.600800 PMC7985460

[28] Jeng CJ, Long CY, Chuang LT. Comparison of magnetic resonance-guided high-intensity focused ultrasound with uterine artery embolization for the treatment of uterine myoma: a systematic literature review and meta-analysis. Taiwan J Obstet Gynecol. 2020;59(5):691–7. 32917320 10.1016/j.tjog.2020.07.012

[29] Babashov V, Palimaka S, Blackhouse G, O’Reilly D. Magnetic resonance-guided high-intensity focused ultrasound (MRgHIFU) for treatment of symptomatic uterine fibroids: an economic analysis. Ont Health Technol Assess Ser. 2015;15(5):1–61. 26357531 PMC4558770

[30] Liu XF, Huang LH, Zhang C, Huang GH, Yan LM, He J. A comparison of the cost-utility of ultrasound-guided high-intensity focused ultrasound and hysterectomy for adenomyosis: a retrospective study. BJOG. 2017;124(Suppl 3):40–5. 28856866 10.1111/1471-0528.14746

[31] Anneveldt KJ, Nijholt IM, Schutte JM, Dijkstra JR, Frederix GWJ, Ista E, et al. Comparison of (cost-)effectiveness of magnetic resonance image-guided high-intensity-focused ultrasound with standard (minimally) invasive fibroid treatments: protocol for a multicenter randomized controlled trial (MYCHOICE). JMIR Res Protoc. 2021;10(11):e29467. 34821569 10.2196/29467 PMC8663707

[32] Ou KY, Jeng CJ, Long CY, Chuang L, Re. A comparison of the cost-utility of ultrasound-guided high-intensity focused ultrasound and hysterectomy for adenomyosis: a retrospective study: is the cost-effectiveness of HIFU for adenomyosis and fibroids feasible? BJOG. 2018;125(6):763–4. 10.1111/1471-0528.15115 29405542

[33] Marinova M, Ghaei S, Recker F, Tonguc T, Kaverina O, Savchenko O, et al. Efficacy of ultrasound-guided high-intensity focused ultrasound (USgHIFU) for uterine fibroids: an observational single-center study. Int J Hyperth. 2021;38(2):30–8. 10.1080/02656736.2021.1939444 34420447

[34] Zhou Y, Zhang J, Chen J, Yang C, Gong C, Li C, et al. Prediction using T2-weighted magnetic resonance imaging-based radiomics of residual uterine myoma regrowth after high-intensity focused ultrasound ablation. Ultrasound Obstet Gynecol. 2022;60(5):681–92. 36054291 10.1002/uog.26053 PMC9828488

[35] Wang YJ, Zhang PH, Zhang R, An PL. Predictive value of quantitative uterine fibroid perfusion parameters from contrast-enhanced Ultrasound for the therapeutic effect of high-intensity focused Ultrasound ablation. J Ultrasound Med. 2019;38(6):1511–7. 30286521 10.1002/jum.14838

[36] Suomi V, Viitala A, Sainio T, Komar G, Sequeiros RB. High-intensity focused Ultrasound Therapy in the uterine fibroid: a clinical case study of poor heating efficacy. Annu Int Conf IEEE Eng Med Biol Soc. 2019;2019:2500–3. 31946405 10.1109/EMBC.2019.8857147

[37] Loring M, Chen TY, Isaacson KB. A systematic review of adenomyosis: it is time to reassess what we thought we knew about the Disease. J Minim Invasive Gynecol. 2021;28(3):644–55. 33371949 10.1016/j.jmig.2020.10.012

[38] Gordts S, Grimbizis G, Campo R. Symptoms and classification of uterine adenomyosis, including the place of hysteroscopy in diagnosis. Fertil Steril. 2018;109(3):380–e81. 29566850 10.1016/j.fertnstert.2018.01.006

[39] Wendel MP, Magann EF. The impact of adenomyosis on pregnancy and pregnancy outcomes: a review. Obstet Gynecol Surv. 2022;77(8):495–500. 35932290 10.1097/OGX.0000000000001042

[40] de Rozario T, Jochum F, Schwaab T, Garbin O, Roy C, Host A. Adenomyosis and obstetric complications: a retrospective case-control study. Eur J Obstet Gynecol Reprod Biol. 2023;292:120–4. 37992424 10.1016/j.ejogrb.2023.11.011

[41] Pados G, Gordts S, Sorrentino F, Nisolle M, Nappi L, Daniilidis A. Adenomyosis and infertility: a Literature Review. Med (Kaunas). 2023;59(9). 10.3390/medicina59091551 PMC10534714 37763670

[42] Jiang L, Han Y, Song Z, Li Y. Pregnancy outcomes after Uterus-sparing operative treatment for adenomyosis: a systematic review and meta-analysis. J Minim Invasive Gynecol. 2023;30(7):543–54. 36972750 10.1016/j.jmig.2023.03.015

[43] Dessouky R, Gamil SA, Nada MG, Mousa R, Libda Y. Management of uterine adenomyosis: current trends and uterine artery embolization as a potential alternative to hysterectomy. Insights Imaging. 2019;10(1):48. 31030317 10.1186/s13244-019-0732-8 PMC6486932

[44] Liu L, Tian H, Lin D, Zhao L, Wang H, Hao Y. Risk of recurrence and Reintervention after Uterine-Sparing interventions for symptomatic adenomyosis: a systematic review and Meta-analysis. Obstet Gynecol. 2023;141(4):711–23. 36897132 10.1097/AOG.0000000000005080 PMC10026977

[45] Li X, Zhu X, He S, Jiang Z, Li H, Tian X, et al. High-intensity focused ultrasound in the management of adenomyosis: long-term results from a single center. Int J Hyperth. 2021;38(1):241–7. 10.1080/02656736.2021.1886347 33602049

[46] Zhu H, Ma Q, Dong G, Yang L, Li Y, Song S, et al. Clinical evaluation of high-intensity focused ultrasound ablation combined with mifepristone and levonorgestrel-releasing intrauterine system to treat symptomatic adenomyosis. Int J Hyperth. 2023;40(1):2161641. 10.1080/02656736.2022.2161641 36586419

[47] Shui L, Mao S, Wu Q, Huang G, Wang J, Zhang R, et al. High-intensity focused ultrasound (HIFU) for adenomyosis: two-year follow-up results. Ultrason Sonochem. 2015;27:677–81. 26050604 10.1016/j.ultsonch.2015.05.024

[48] Xiong Y, Yue Y, Shui L, Orsi F, He J, Zhang L. Ultrasound-guided high-intensity focused ultrasound (USgHIFU) ablation for the treatment of patients with adenomyosis and prior abdominal surgical scars: a retrospective study. Int J Hyperth. 2015;31(7):777–83. 10.3109/02656736.2015.1071436 26367457

[49] Zhang L, Rao F, Setzen R. High intensity focused ultrasound for the treatment of adenomyosis: selection criteria, efficacy, safety and fertility. Acta Obstet Gynecol Scand. 2017;96(6):707–14. 28437003 10.1111/aogs.13159

[50] Cheung VY. Current status of high-intensity focused ultrasound for the management of uterine adenomyosis. Ultrasonography. 2017;36(2):95–102. 28145109 10.14366/usg.16040 PMC5381845

[51] Li W, Mao J, Liu Y, Zhu Y, Li X, Zhang Z, et al. Clinical effectiveness and potential long-term benefits of high-intensity focused ultrasound therapy for patients with adenomyosis. J Int Med Res. 2020;48(12):300060520976492. 33349096 10.1177/0300060520976492 PMC7758569

[52] Fan TY, Zhang L, Chen W, Liu Y, He M, Huang X, et al. Feasibility of MRI-guided high intensity focused ultrasound treatment for adenomyosis. Eur J Radiol. 2012;81(11):3624–30. 21719223 10.1016/j.ejrad.2011.05.036

[53] Zhou M, Chen JY, Tang LD, Chen WZ, Wang ZB. Ultrasound-guided high-intensity focused ultrasound ablation for adenomyosis: the clinical experience of a single center. Fertil Steril. 2011;95(3):900–5. 21067723 10.1016/j.fertnstert.2010.10.020

[54] Marques ALS, Andres MP, Kho RM, Abrao MS. Is high-intensity focused Ultrasound Effective for the Treatment of Adenomyosis? A systematic review and Meta-analysis. J Minim Invasive Gynecol. 2020;27(2):332–43. 31377454 10.1016/j.jmig.2019.07.029

[55] Gong C, Wang Y, Lv F, Zhang L, Wang Z. Evaluation of high intensity focused ultrasound treatment for different types of adenomyosis based on magnetic resonance imaging classification. Int J Hyperth. 2022;39(1):530–8. 10.1080/02656736.2022.2052366 35300545

[56] Feng Y, Hu L, Chen W, Zhang R, Wang X, Chen J. Safety of ultrasound-guided high-intensity focused ultrasound ablation for diffuse adenomyosis: a retrospective cohort study. Ultrason Sonochem. 2017;36:139–45. 28069193 10.1016/j.ultsonch.2016.11.022

[57] Haiyan S, Lin W, Shuhua H, Wang W. High-intensity focused ultrasound (HIFU) combined with gonadotropin-releasing hormone analogs (GnRHa) and levonorgestrel-releasing intrauterine system (LNG-IUS) for adenomyosis: a case series with long-term follow up. Int J Hyperth. 2019;36(1):1179–85. 10.1080/02656736.2019.1679892 31793356

[58] Guo Q, Xu F, Ding Z, Li P, Wang X, Gao B. High intensity focused ultrasound treatment of adenomyosis: a comparative study. Int J Hyperth. 2018;35(1):505–9. 10.1080/02656736.2018.1509238 30306813

[59] Guo Y, Duan H, Cheng J, Zhang Y. Gonadotrophin-releasing hormone agonist combined with high-intensity focused ultrasound ablation for adenomyosis: a clinical study. BJOG. 2017;124(Suppl 3):7–11. 28856862 10.1111/1471-0528.14736

[60] Pang LL, Mei J, Fan LX, Zhao TT, Li RN, Wen Y. Efficacy of high-intensity focused Ultrasound Combined with GnRH-a for adenomyosis: a systematic review and Meta-analysis. Front Public Health. 2021;9:688264. 34485218 10.3389/fpubh.2021.688264 PMC8415267

[61] Ye MZ, Deng XL, Zhu XG, Xue M. [Clinical study of high intensity focused ultrasound ablation combined with GnRH-a and LNG-IUS for the treatment of adenomyosis]. Zhonghua Fu Chan Ke Za Zhi. 2016;51(9):643–9. 27671043 10.3760/cma.j.issn.0529-567X.2016.09.002

[62] Xu Y, Zhou Z, Wang H, Shao L, Liu G. High-intensity focused Ultrasound Combined with Gonadotropin-releasing hormone agonist or levonorgestrel-releasing Intrauterine System in treating dysmenorrhea of severe adenomyosis. J Comput Assist Tomogr. 2021;45(2):224–31. 33661158 10.1097/RCT.0000000000001138

[63] Zhao TT, Pang LL, Yang LL, Li RN, Fan LX, Wen Y. Efficacy of high-intensity focused ultrasound combined with LNG-IUS for adenomyosis: a systematic review and meta-analysis. Arch Gynecol Obstet. 2022. 10.1007/s00404-022-06720-z 35947146

[64] Liu X, Wang W, Wang Y, Wang Y, Li Q, Tang J. Clinical predictors of long-term success in Ultrasound-guided high-intensity focused ultrasound ablation treatment for adenomyosis: a retrospective study. Med (Baltim). 2016;95(3):e2443. 10.1097/MD.0000000000002443 PMC4998251 26817877

[65] Machtinger R, Inbar Y, Cohen-Eylon S, Admon D, Alagem-Mizrachi A, Rabinovici J. MR-guided focus ultrasound (MRgFUS) for symptomatic uterine fibroids: predictors of treatment success. Hum Reprod. 2012;27(12):3425–31. 23019304 10.1093/humrep/des333

[66] Keserci B, Duc NM. The role of T1 perfusion-based classification in predicting the outcome of magnetic resonance-guided high-intensity focused ultrasound treatment of adenomyosis. Int J Hyperth. 2018;34(3):306–14. 10.1080/02656736.2017.1326634 28540825

[67] Gu J, Lin B, Guo Z, Aili A. How to boost an obstetrician’s confidence in vaginal delivery after high-intensity focused ultrasound: a comparison study on delivery outcomes. Int J Hyperth. 2022;39(1):900–6. 10.1080/02656736.2022.2083700 35848403

[68] Gong C, Yang B, Shi Y, Liu Z, Wan L, Zhang H, et al. Factors influencing the ablative efficiency of high intensity focused ultrasound (HIFU) treatment for adenomyosis: a retrospective study. Int J Hyperth. 2016;32(5):496–503. 10.3109/02656736.2016.1149232 27385316

[69] Gong C, Setzen R, Liu Z, Liu Y, Xie B, Aili A, et al. High intensity focused ultrasound treatment of adenomyosis: the relationship between the features of magnetic resonance imaging on T2 weighted images and the therapeutic efficacy. Eur J Radiol. 2017;89:117–22. 28267526 10.1016/j.ejrad.2017.02.001

[70] Du CC, Wang YQ, Qu DC, Jiang LP, Yu X, Zhou HG. Magnetic resonance imaging T2WI hyperintense foci number and the prognosis of adenomyosis after high-intensity focused ultrasound treatment. Int J Gynaecol Obstet. 2021;154(2):241–7. 33421123 10.1002/ijgo.13587

[71] Tang Y, Ming-Tao Y, Xiang RM, Xu W, Zhang RY, Weng MB, et al. Preoperative CA125 as a risk factor for symptom recurrence of adenomyosis after ultrasound-guided high-intensity focused ultrasound ablation surgery. Int J Hyperth. 2022;39(1):1164–9. 10.1080/02656736.2022.2107716 36075579

[72] Rezk A, Kahn J, Singh M. Fertility Sparing Management in Uterine fibroids. StatPearls. Treasure Island (FL) ineligible companies. Disclosure: Jenna Kahn declares no relevant financial relationships with ineligible companies. Disclosure: Manvinder Singh declares no relevant financial relationships with ineligible companies.2024.

[73] Gulino FA, Dilisi V, Capriglione S, Cannone F, Catania F, Martire FG, et al. Anti-mullerian hormone (AMH) and adenomyosis: mini-review of literature of the last 5 years. Front Endocrinol (Lausanne). 2022;13:1014519. 36120472 10.3389/fendo.2022.1014519 PMC9471373

[74] Lee JS, Hong GY, Lee KH, Kim TE. Changes in anti-mullerian hormone levels as a biomarker for ovarian reserve after ultrasound-guided high-intensity focused ultrasound treatment of adenomyosis and uterine fibroid. BJOG. 2017;124(Suppl 3):18–22. 28856867 10.1111/1471-0528.14739

[75] Laughlin-Tommaso S, Barnard EP, AbdElmagied AM, Vaughan LE, Weaver AL, Hesley GK, et al. FIRSTT study: randomized controlled trial of uterine artery embolization vs focused ultrasound surgery. Am J Obstet Gynecol. 2019;220(2):174. e1- e13. 10.1016/j.ajog.2018.10.032 PMC6355152 30696556

[76] Cui Y, Dong Y, Guo B, Xing C, Gao X, Su D. Effect of HIFU on endometrial receptivity and sex hormone level in uterine fibroid patients and analysis of influencing factors for its treatment rate. Exp Ther Med. 2019;17(3):2291–7. 30867713 10.3892/etm.2019.7194 PMC6395971

[77] Liu X, Xue L, Wang Y, Wang W, Tang J. Vaginal delivery outcomes of pregnancies following ultrasound-guided high-intensity focused ultrasound ablation treatment for uterine fibroids. Int J Hyperth. 2018;35(1):510–7. 10.1080/02656736.2018.1510548 30354861

[78] Li JS, Wang Y, Chen JY, Chen WZ. Pregnancy outcomes in nulliparous women after ultrasound ablation of uterine fibroids: a single-central retrospective study. Sci Rep. 2017;7(1):3977. 28638108 10.1038/s41598-017-04319-y PMC5479832

[79] Zou M, Chen L, Wu C, Hu C, Xiong Y. Pregnancy outcomes in patients with uterine fibroids treated with ultrasound-guided high-intensity focused ultrasound. BJOG. 2017;124(Suppl 3):30–5. 28856864 10.1111/1471-0528.14742

[80] Huang X, Yu D, Zou M, Wang L, Xing HR, Wang Z. The effect of exercise on high-intensity focused ultrasound treatment efficacy in uterine fibroids and adenomyosis: a retrospective study. BJOG. 2017;124(Suppl 3):46–52. 28856860 10.1111/1471-0528.14748

[81] Anneveldt KJ, van ‘t Oever HJ, Nijholt IM, Dijkstra JR, Hehenkamp WJ, Veersema S, et al. Systematic review of reproductive outcomes after high intensity focused Ultrasound treatment of uterine fibroids. Eur J Radiol. 2021;141:109801. 34116454 10.1016/j.ejrad.2021.109801

[82] Wu G, Li R, He M, Pu Y, Wang J, Chen J, et al. A comparison of the pregnancy outcomes between ultrasound-guided high-intensity focused ultrasound ablation and laparoscopic myomectomy for uterine fibroids: a comparative study. Int J Hyperth. 2020;37(1):617–23. 10.1080/02656736.2020.1774081 32525708

[83] Li W, Yang Z, Gao B, Zou L, Xu D, Liu L, et al. Comparison of ultrasound-guided high-intensity focused ultrasound ablation and hysteroscopic myomectomy for submucosal fibroids: a retrospective study. Int J Hyperth. 2021;38(1):1609–16. 10.1080/02656736.2021.1995053 34763580

[84] Wei J, Wang L, Tao H, Wang X, Zheng F, He P, et al. Comparison of pregnancy outcomes in infertile patients with different types of adenomyosis treated with high-intensity focused ultrasound. Int J Hyperth. 2023;40(1):2238140. 10.1080/02656736.2023.2238140 37495217

[85] Zhou CY, Xu XJ, He J. [Pregnancy outcomes and symptom improvement of patients with adenomyosis treated with high intensity focused ultrasound ablation]. Zhonghua Fu Chan Ke Za Zhi. 2016;51(11):845–9. 27916069 10.3760/cma.j.issn.0529-567X.2016.11.009

[86] Huang YF, Deng J, Wei XL, Sun X, Xue M, Zhu XG, et al. A comparison of reproductive outcomes of patients with adenomyosis and infertility treated with high-intensity focused ultrasound and laparoscopic excision. Int J Hyperth. 2020;37(1):301–7. 10.1080/02656736.2020.1742390 32208771

[87] Ou KY, Jeng CJ, Long CY, Chuang L, Re. Pregnancy outcomes in patients with uterine fibroids treated with ultrasound-guided high-intensity focused ultrasound: is the noninvasive nature of HIFU ablation for uterine fibroids and adenomyosis setting patients up for future operative delivery? BJOG. 2018;125(6):762–3. 10.1111/1471-0528.15114 29405613

[88] Kim TE. Author’s reply re: changes in anti-mullerian hormone levels as a biomarker for ovarian reserve after ultrasound-guided high-intensity focused ultrasound treatment of adenomyosis and uterine fibroid. BJOG. 2018;125(4):508–9. 29148166 10.1111/1471-0528.14979

[89] Li XW, Liang MY, Wang JL, Wang DP. Spontaneous uterine rupture during late pregnancy after high-intensity focused Ultrasound. Chin Med J (Engl). 2015;128(10):1419. 25963369 10.4103/0366-6999.156819 PMC4830328

[90] Lai THT, Seto MTY, Cheung VYT. Intrapartum uterine rupture following ultrasound-guided high-intensity focused ultrasound ablation of uterine fibroid and adenomyosis. Ultrasound Obstet Gynecol. 2022;60(6):816–7. 35748875 10.1002/uog.24983

[91] Liu Y, Fu N, Lv B, He Y, Wang X. Uterine rupture after high-intensity focused ultrasound ablation of adenomyosis: a case report and literature review. Int J Hyperth. 2023;40(1):2212885. 10.1080/02656736.2023.2212885 37217194

